# Co-designing a Sexual Health App With Immigrant Adolescents: Protocol for a Qualitative Community-Based Participatory Action Research Study

**DOI:** 10.2196/45389

**Published:** 2023-03-22

**Authors:** Salima Meherali, Sarah Munro, Giulia Puinean, Bukola Salami, Josephine Pui-Hing Wong, Ashley Vandermorris, James Russell Andrew Benoit, Sarah Flicker, Philomina Okeke-Ihejirika, Eleni Stroulia, Wendy V Norman, Shannon D Scott

**Affiliations:** 1 University of Alberta Edmonton, AB Canada; 2 University of British Columbia Vancouver, BC Canada; 3 Toronto Metropolitan University Toronto, ON Canada; 4 University of Toronto Toronto, ON Canada; 5 York University Toronto, ON Canada

**Keywords:** adolescent health, health promotion, immigrants, information needs, mHealth, sexual and reproductive health, access to care

## Abstract

**Background:**

Canada is one of the world’s most ethnically diverse countries, with over 7 million individuals out of a population of 38 million being born in a foreign country. Immigrant adolescents (aged 10 to 19 years) make up a substantial proportion of newcomers to Canada. Religious and cultural practices can influence adolescents’ sexual attitudes and behaviors, as well as the uptake of sexual and reproductive health (SRH) services among this population. Adolescence is a time to establish lifelong healthy behaviors. Research indicates an alarming gap in adolescents’ SRH knowledge, yet there is limited research on the SRH needs of immigrant adolescents in Canada.

**Objective:**

The purpose of this study is to actively engage with immigrant adolescents to develop, implement, and evaluate a mobile health (mHealth) intervention (ie, mobile app). The interactive mobile app will aim to deliver accurate and evidence-based SRH information to adolescents.

**Methods:**

We will use community-based participatory action research to guide our study. This research project will be conducted in 4 stages based on user-centered co-design principles. In Stage 1 (Empathize), we will recruit and convene 3 adolescent advisory groups in Edmonton, Toronto, and Vancouver. Members will be engaged as coresearchers and receive training in qualitative and quantitative methodologies, sexual health, and the social determinants of health. In Stage 2 (Define and Ideate), we will explore SRH information and service needs through focus group discussions with immigrant adolescents. In Stage 3 (Prototype), we will collaborate with mobile developers to build and iteratively design the app with support from the adolescent advisory groups. Finally, in Stage 4 (Test), we will return to focus group settings to share the app prototype, gather feedback on usability, and refine and release the app.

**Results:**

Recruitment and data collection will be completed by February 2023, and mobile app development will begin in March 2023. The mHealth app will be our core output and is expected to be released in the spring of 2024.

**Conclusions:**

Our study will advance the limited knowledge base on SRH and the information needs of immigrant adolescents in Canada as well as the science underpinning participatory action research methods with immigrant adolescents. This study will address gaps by exploring SRH priorities, health information needs, and innovative strategies to improve the SRH of immigrant adolescents. Engaging adolescents throughout the study will increase their involvement in SRH care decision-making, expand efficiencies in SRH care utilization, and ultimately improve adolescents' SRH outcomes. The app we develop will be transferable to all adolescent groups, is scalable in international contexts, and simultaneously leverages significant economies of scale.

**International Registered Report Identifier (IRRID):**

PRR1-10.2196/45389

## Introduction

### Background

Canada is one of the world’s most ethnically diverse countries, with over 7 million individuals born in a foreign country [1]. Globally, Canada has one of the highest percentages of immigrants, who comprise 21.9% of the total population [2]. Of all these newcomers, approximately 34% are under the age of 25, with over 60% of immigrant youth coming from Asia, the Middle East, and Africa [1]. Given these statistics, understanding health care service information needs and utilization by immigrant adolescents is vital for health care planning, policy, and delivery. Adolescence is a critical period in the transition from childhood into adulthood, during which young individuals aged 10 to 19 years undergo substantial physical, psychological, social, and emotional changes [3]. Adolescents are a vulnerable population because of their age-related psychosocial and biological changes and the challenges associated with navigating these changes [4]. As part of this development, it is common for adolescents to explore their sexual identities and feelings through sexual experimentation [5]. Depending on their knowledge about sexuality and social support, some may experience unintended consequences, such as unplanned pregnancy and sexually transmitted infections (STIs). Neglect of adolescents’ sexual and reproductive health (SRH) needs can affect their physical and mental health, future employment, economic well-being, and ability to reach their full potential [6-8]. A small but growing body of literature suggests that immigrant youth lack SRH knowledge, use fewer sexual health services, and receive fewer sex education resources than nonimmigrant youth [9-11]. This difference appears to be heavily influenced by religious and cultural practices [12]. Immigrant families from Southeast Asia and the Middle East have diverse cultural and religious views on dating, relationships, adolescent sexuality, gender roles and identity, and sexual orientation [13]. Studies have shown that many immigrant adolescents find it challenging to balance family expectations, cultural standards, and religious demands; dissonance can cause internal and external conflict [13-15]. In addition, many adolescents experience intergenerational conflict, a competition between their own values and those of elders of the community [16].

Despite the large number of immigrant adolescents in Canada, there is limited research on the SRH of this population. COVID-19 pandemic–driven changes in how services are provided, such as limiting in-person care and deferring new and walk-in patients, have created barriers for adolescents to access confidential SRH information and services. Individuals may want to avoid obtaining in-person care during this time to reduce the risk of viral transmission. Hence, it is imperative to promote and adopt innovative approaches such as digital health (mobile apps, information through SMS text messaging) to deliver culturally appropriate SRH and sexuality education to immigrant adolescents in Canada. Beyond pandemic concerns, there is evidence to suggest that mobile apps have the potential to promote adolescent health [17]. This study will address these gaps by exploring the SRH priorities and health information needs of immigrant adolescents and will develop an interactive sexual health promotion mobile app for immigrant adolescents in Canada.

### SRH Needs of Adolescents

The transition from adolescence to adulthood is complex, as new responsibilities materialize and conﬁdence and independence emerge [18,19]. Yet, teens often have inadequate knowledge about the consequences of unprotected sex and are novices when it comes to implementing safe sex practices. These consequences include unwanted pregnancy, complications of unsafe abortion, and STIs [11]. In addition, due to fear, stigma, and confidentiality issues, many do not reveal their SRH problems and underutilize the health care services they need [9]. SRH is an important aspect of adolescent health and well-being, and it interconnects with various physical and mental health issues [20-22].

Globally, the SRH needs of adolescents have been largely unmet and pose a significant threat to the health and well-being of adolescents [9,23-25]. They are biologically more vulnerable to infections, more susceptible to peer pressure, developmentally more disposed to risk-taking, and lack the skills and confidence to negotiate safer sexual practices. STI rates among Canadian adolescents are on the rise [24-28]. Studies on adolescent SRH have found that the unintended teenage pregnancy rate is rising again in Canada [29]. The Canadian Community Health Survey reported that almost 15.5% of Canadian youth are at high risk of unintended pregnancy due to the nonuse of contraceptives [30,31]. The consequences of teenage pregnancy include anemia, hypertension, renal disease, eclampsia, and depressive disorders [29]. Research has found that adolescent mothers experience more mental health problems and higher rates of postpartum depression than women aged 25 and older [32]. Moreover, children of teenagers are more likely to have low birth weights and experience associated health problems [29]. Studies highlight an alarming gap in the sexual health knowledge of adolescents in Canada despite their completion of sexual education requirements in school [27,33-36]. Studies also found that sexually active Canadian teenagers commonly engage in risky sexual behaviors, including unprotected sex, multiple sexual partners, and intercourse before 15 years of age [35,37,38]. Moreover, many Canadian youths do not consider themselves at risk for HIV/AIDS, and their overall knowledge base about the disease has declined over the last 2 decades [9,39,40].

### Mobile Phone–Based App to Increase Awareness and Uptake of SRH Services

Mobile app platforms have the potential to advance SRH. Nearly 100% of youth in Canada aged 15 to 24 years use the internet daily or own a smartphone [41]. The use of mobile technology for health promotion offers privacy [42-47], access to personalized information [42,44,46,48], and convenience [42,47,49], making it a valuable way to provide accurate information about sexual health to adolescents [43-48]. Furthermore, young people are responsive to and excited about using new technologies for SRH promotion [42,45,50,51]. Innovative ways to deliver sexual health information are more important than ever before due to the impact of the COVID-19 pandemic on access to SRH services globally [52]. The absence of adolescent SRH services from “essential” health services during COVID-19 amplifies the potential role of technology to meet the SRH needs of vulnerable groups like adolescents. Amid the pandemic, technology has largely provided the means to support the recalibration of health systems, service provision, and information delivery [53]. Just as digital behavior changes, campaigns on hand washing, social distancing, and wearing masks have supported the pandemic response, we can digitally connect with young people to support their access to SRH information and services during times of social isolation and crisis. Although there are many mobile apps addressing SRH worldwide, we could not locate any research on co-designing a mobile app to improve immigrant adolescent SRH in Canada. Research has demonstrated that access to socially inclusive and culturally relevant SRH information significantly improves SRH knowledge and communication skills with partners and parents, improves contraceptive knowledge and use, and reduces risk-taking sexual behaviors by young people [15,27,54]. Therefore, the aim of this study is to engage immigrant adolescents in developing, implementing, and evaluating a mobile health (mHealth) app that will deliver evidence-based SRH information to this population.

## Methods

### Theoretical Framework

Intersectionality places an explicit focus on differences among people and seeks to illuminate various interacting social factors that affect human lives, including social locations, health status, and quality of life [55]. Intersectionality asserts that people are often disadvantaged by multiple interlocking sources of oppression (ie, their race, class, gender identity, sexual orientation, religion, and other identity markers) [56]. The intersection of race, gender, age, and socioeconomic status may pose barriers to young immigrant populations seeking SRH education and services. This framework will help us understand these differences, considering gender, race, class, sexual orientation, and migration histories, as they impact immigrant adolescents seeking SRH services.

### Study Design

A community-based participatory action research (CBPAR) framework will guide our study [57-61]. CBPAR is a context-specific process driven by participants that requires innovative methodological approaches [62,63]. The principal outcome of this adolescent-partnered project is to develop an mHealth intervention that responds to SRH needs. Incorporating the end users’ voices into the design and development of these interventions is crucial, as it has been noted that few mobile interventions are truly effective and scalable [64-66]. A user-centered co-design approach will increase the likelihood that adolescents will use the app and that it will be scalable and transferable to other contexts. Our goal for this engagement approach is to create an environment that supports the metacriterion of respect, trust, legitimacy, fairness, competence, and accountability in the development of knowledge [66]. This project will have 4 stages based on user-centered co-design principles [67].

### Key Stages of the Research Project

#### Stage 1: Empathize

For a user-centered design, it is important that we engage the people for whom we are planning to design the product. Therefore, we will first develop and convene adolescent advisory groups (AAGs) in Edmonton, Toronto, and Vancouver. These cities have the highest percentages of immigrant populations from all geographic locations [2,68]. Having a multisite project will help ensure that the app will reach and be disseminated to immigrant adolescents across Canada. The AAGs will participate actively throughout the project (ie, in data collection, data analysis, mobile app development, gathering feedback on app usability, and knowledge mobilization). AAGs will be informed through our national environmental scan of youth advisory groups [69]. To recruit adolescents for the AAGs, we will hire 3 research assistants (RAs) (one at each site) who are skilled in research methods and have substantial youth facilitation expertise and experience working with diverse communities. Peer RAs will also collaborate with RAs to facilitate the recruitment of participants for AAGs. AAG members will be recruited from immigrant communities, public agencies, community and recreation centers, and immigrant service agencies. Participants aged 12 to 19 years from diverse ethnic backgrounds will be invited to participate in the AAG. Based on the developmental stage of young people, particularly in terms of physiological, psychological, and social development processes, we anticipate that it might be hard for younger adolescents to share common “cultural” beliefs and values. In addition, older teens may not allow young adolescents to share their thoughts and may dismiss younger teens. To provide equal opportunity for all to participate, we will create 2 separate groups. Adolescents aged 12 to 15 years will be placed in 1 group, and adolescents aged 16 to 19 years will be in a separate group. We anticipate that a reasonable target size of between 8 and 12 adolescents will create meaningful engagement [65,69] and that we will meet for 2 hours via Zoom or Google Meet every 2 months for approximately 18 to 24 months. To foster meaningful engagement, AAG members will receive training in qualitative and quantitative methodologies, sexual health, and the social determinants of health (see Multimedia Appendix 1).

#### Stage 2: Define and Ideate

##### Overview

To explore the experiences, information needs, and challenges of immigrant adolescents related to SRH and how to address these challenges, we will conduct qualitative individual interviews or focus groups (FGs) following the CBPAR methodology [59,60]. SRH is a sensitive topic, and adolescents sometimes feel uncomfortable discussing their SRH needs in front of others. Participants comfortable with FGs will be invited to FGs. If participants are not comfortable, they will be invited for individual interviews.

##### Participants

We will recruit immigrant adolescents aged 12 to 19 years who were born in a country other than Canada and who immigrated in the last 10 years or are part of a second-generation cohort. We will focus on this age group because we aim to understand the factors in early and late adolescence that predispose young people to subsequent sexual health risks. In addition, adolescents aged 12 to 19 years are generally mature enough to participate in individual interviews or FGs [70-72].

We will use a multifaceted, community-based strategy to recruit immigrant adolescents from diverse ethnic groups. Public, community, and immigrant service agencies will support participant recruitment. In addition, social marketing campaigns (eg, Instagram advertising as well as posting of study ads by youth and health-oriented organizations) and snowball sampling will be used to recruit participants. Peer RAs and graduate RAs hired as part of this project will also assist with recruitment. We will also include non-English speaking participants. Our peer RAs are bilingual, and we will hire multilingual graduate RAs for this project who speak Canada’s most common languages other than English to provide safe interpretation services for participants who do not speak English fluently.

We will conduct between 20 and 25 individual interviews or 3 to 4 FGs in each city. The participants for FGs will be divided into 2 groups (12 to 15 years and 16 to 19 years) to ensure that our data also capture the SRH needs of younger teens. The individual interviews or FGs will occur in person or over Zoom. The final sample size will be based on data saturation [73]. Individual interviews and FGs will be consistent in their delivery. Discussion topics will focus on issues related to SRH, sexual orientation, gender identity, sexual health history, dating and relationship experiences, source of information about SRH, cultural relevance and trustworthiness of those sources, challenges to receiving SRH information and services, and familiarity with SRH mobile apps and websites. We will conduct separate FGs for male and female participants and a mixed-gender FG based on participants’ comfort and availability. Transcripts will be digitally recorded and transcribed verbatim by a professional.

##### Data Analysis

In accordance with CBPAR, we will use the DEPICT (dynamic reading, engaged codebook development, participatory coding, inclusive reviewing and summarizing of categories, collaborative analyzing, and translating) model for participatory analysis (Multimedia Appendix 2) [74]. Data analysis will occur in four stages: (1) conversations will be digitally recorded and transcribed verbatim by a professional transcriptionist; (2) 2 graduate RAs, peer RAs, and 2 members of the research team will read transcripts in detail several times; (3) the nominated principal applicant and the principal applicant will lead the team in conducting open coding of all transcripts and then group codes into preliminary themes using thematic analysis [75]. The themes will be presented to the larger investigator team and AAGs for feedback and additional comments, which will then be incorporated into the analysis; and (4) themes across cases will be grouped into an organizational framework. To achieve reliability and validity and to ensure rigor, we will employ verification strategies that will identify when to continue, stop, or modify the research process. Verification strategies will include (1) methodological coherence, ensuring congruence between the research questions; (2) appropriate sampling to ensure efficient and effective saturation of categories with optimal quality; (3) collecting and analyzing data concurrently; (4) developing a coding system that will be discussed and verified with research team members and AAG members; (5) keeping a detailed audit trail and field notes [76]; and (6) member checks. Then, we will share the transcripts, results, analyses, and reports with participants so they can review and provide feedback on the results and analyses. NVivo software (version 12; QSR International) will be used to manage the data analysis process.

#### Stage 3: Prototype

Informed by the Stage 2 findings and with integrated, continuous input from the AAGs, we will develop a prototype of a mobile app to house SRH content. The content of the app will stem from two primary sources: (1) evidence-based resources such as the Society of Obstetricians and Gynecologists of Canada, the Public Health Agency of Canada, and the World Health Organization; and (2) themes and experiences generated from individual interviews and FGs in Stage 2. The app will include age-specific information separately so that adolescents of different age groups can easily navigate the information related to them. In addition, information related to accessing SRH services will also be provided to connect adolescents to SRH services in their community.

The AAGs and peer RAs will work with the app developer to iteratively revise and build the app to address questions of clarity, potential sources of bias or marginalizing design elements, ease of use, relevance, and other factors determined by the AAGs. The app will use an internal analytics system to run analysis on user demographics, feedback, and content views to gather information on how the app is being used. The app content will be approved by team members who have expertise in SRH to ensure evidence accuracy and appropriate evidence interpretation. We will initially develop the interventions in English only to consolidate the findings and best practice functionality. Once the app has demonstrated usability and effectiveness in English, we plan to apply for new funding to translate the app content into languages used by immigrant communities.

#### Stage 4: Usability Testing of Mobile App Prototype

At the end of the project, we will run several FGs to evaluate the usability, acceptability, and effectiveness of the app; seek further input for refining it; evaluate the effectiveness of the project; and seek direction for future research. For usability testing of the app, we will conduct (1) 6 FGs with 5 to 10 immigrant adolescent participants (n=30-60) in Edmonton, Toronto, and Vancouver (2 FGs in each city) and (2) 3 FGs with 5 to 8 service providers, policy makers, and members of the communities’ informal support channels (n=15-20).

At the start of each FG, participants will be asked to download the app and use it on their own. Then, the researcher will begin the FG. Semistructured FGs will focus on questions about the app informed by a systematic search of over 180 usability evaluations [77]: participants’ views, their preferences regarding receipt of health information, useful attributes, unhelpful elements, perceptions of the utility to improve adolescent’s SRH, and recommended revisions and additions. Each aspect of the app (eg, narrative, visual appeal, audio appeal, health information, engagement, and interactivity) will be explored in the FGs. Interviews will be audio recorded, transcribed verbatim, and thematically analyzed [75]. We will integrate these findings into the app. The final version will then be disseminated using established social media platforms, such as TikTok, Twitter, and Instagram. The AAG members and peer RAs will participate in dissemination activities. The app will be available for free download on the Apple App Store and Google Play. In the future, we will conduct pragmatic trials to evaluate the app’s effectiveness in improving SRH outcomes for adolescents.

### App Sustainability

This study will develop a holistic, prescriptive model that can be used to ensure the sustainability and scalability of mHealth apps in the public health care sector in Canada and other countries. Our long-term community partners in all 3 cities will support the sustainability of the app. These organizations will host the app, as they need an evidence-based tool to help immigrant adolescents increase their awareness and uptake of SRH services to improve SRH outcomes.

### Ethics Approval

This study received ethics approval from the University of Alberta Ethics Board (Pro0013664).

## Results

Recruitment and data collection will be completed by February 2023, and we will begin co-designing the mobile app in March 2023. We expect to share a prototype of the app, gather feedback on its usability, and then refine and release the app in the spring of 2024. We will engage immigrant adolescents and service providers as partners in the development of the mobile app to ensure that immigrant adolescents are aware of this tool and will use the app, helping contribute to positive SRH outcomes. This study will also evaluate the usability of the mobile app. In the future, we will conduct pragmatic trials to evaluate the effectiveness of the app in improving the SRH outcomes of adolescents.

## Discussion

### Expected Findings

This study aims to develop a mobile SRH app designed by and targeted to immigrant adolescents in Canada. Our exciting and novel adolescent-engaged methods and mobile app have the potential to rapidly address the unmet SRH needs of immigrant adolescents and to improve the SRH of immigrant adolescents across Canada. This app is important because it allows immigrant adolescents to independently access resources related to SRH and mitigates the impact of familial bias on teens’ ability to seek out such information. Further, young people are very comfortable utilizing mobile apps. This engaging modality will make it easier for immigrant adolescents to learn about safe SRH practices and may support them in having the knowledge and motivation to access services. The expected outputs of this study include:

- We will be able to identify the SRH needs of an underresearched population (ie, immigrant adolescents) in Canada.
- Voices of the full spectrum of gender diversity (including transgender and gender nonconforming adolescents), cultural knowledge, and agency will inform the mobile app.
- An innovative, adolescent-centered knowledge translation tool (ie, the mobile app) will be developed to help improve the SRH of immigrant adolescents in Canada.

The potential impact of this mobile app development includes increasing SRH knowledge and awareness, access to SRH services, and the use of contraception while decreasing unintended pregnancies and the need for abortion among adolescents. Our overall program of research is to build upon the active engagement of immigrant youth to develop, evaluate, and implement innovative and evidence-based digital knowledge translation tools and to build the resilience that immigrant youth need to successfully integrate into Canadian society. This research epitomizes creativity and innovation by merging the arts, sciences, and the engagement of youth in research.

### Conclusion

Targeting adolescents and using engaging modalities has a strong potential to effectively increase their involvement in SRH care decision-making, expand efficiencies in SRH care utilization, and ultimately improve adolescents’ SRH outcomes. We will disseminate the mobile app, along with other findings of our study, via community symposiums; public training sessions in Edmonton, Toronto, and Vancouver; and a website hosted by the University of Alberta Faculty of Nursing. Our findings will also be presented at academic conferences and published in open-access, peer-reviewed journals, advancing the body of knowledge on immigrant adolescents’ SRH needs.

## Appendix

Multimedia Appendix 1 Adolescent advisory group training activities.

Multimedia Appendix 2 DEPICT steps, roles, and guiding questions.

Multimedia Appendix 3 Canadian Institutes of Health Research / Instituts de recherche en santé du Canada (CIHR/IRSC) - Operating Grant: Early Career Investigator Grants in Maternal, Reproductive, Child & Youth Health/Subv. de fonct.: Subventions de chercheurs en début de carrière en santé maternelle, en santé reproductive, et en santé des enfants/adolescents - Committee/Comité: Operating Grant: New Investigator Grants in Maternal, Reproductive, Child & Youth Health/Sub. de nouveaux chercheurs en santé maternelle, santé reproductive, et enfants/adolescents (Canada).

## Abbreviations

AAG: adolescent advisory group
CBPAR: community-based participatory action research
DEPICT: dynamic reading, engaged codebook development, participatory coding, inclusive reviewing and summarizing of categories, collaborative analyzing, translating
FG: focus group
mHealth: mobile health
RA: research assistant
SRH: sexual and reproductive health
STI: sexually transmitted infection

## Data Availability

The data generated during this study are not publicly available yet because data collection is ongoing, and results have not yet been analyzed. However, data collection materials are available from the corresponding author upon reasonable request.
