# Supplementary material for: Co-designing a Sexual Health App With Immigrant Adolescents: Protocol for a Qualitative Community-Based Participatory Action Research Study
Source: JMIR Res Protoc. 2023 Mar 22;12:e45389. doi: 10.2196/45389 (PMC10131995; doi:10.2196/45389)
Supplement: Multimedia Appendix 1 [file resprot_v12i1e45389_app1.docx]

**Supplementary file 1**

**Adolescent Advisory Group Training Activities**

(Subject to change based on AAG members’ needs)

**Information about training:**

The training for the adolescent advisory group (AAG) includes a mix of group training sessions and individual study/practice. There will be 7 separate group training sessions. Training sessions range in length from 1.5- 2 hours and will all take place virtually on Zoom or Google Meet. The training sessions will be led by NPA, PA, graduate research assistants and peer research assistants. The training sessions will include group discussions and opportunities to inform the actual study. While the facilitators will be there to provide the AAG with important information, there will also be an opportunity for all to learn together and to improve the study as a team. AAG group members will be encouraged to ask questions, provide feedback and comments, and join in the discussions. At the end of this training if AAG members feel that there are things we have missed or there are some topics they would like more information on, there will be an opportunity for them to inform the facilitators and we will explore different options for addressing those needs.

**Training Schedule:**

**Training Session 1: Study Operations**

Date/Time: TBD

**Topics Covered:**

- Welcome to training session #1 – What to expect from your training experience.
- The AAG position (roles and responsibilities).
- The study team structure (members and meetings).
- Important terms & definitions.
- Participant privacy and confidentiality forms.

**Training Session 2: Adolescent sexual and reproductive health**

Date/Time: TBD

**Topics Covered:**

- Ice breaker.
- What is sexual health and right.
- Why knowledge about sexual health and right is important for adolescents.
- What are the challenges adolescents face related to sexual and reproductive health and right?
- Consequences of lack of knowledge about SRHR on adolescent’s health and over all well-being.
- Share the previous scoping review and qualitative study findings conducted by NPA.
- How the project will help to understand the adolescents SRH information needs?

**Training Session 3: Qualitative Research**

Date/Time: TBD

**Topics Covered:**

• Introductions and ice breaker

• Review from training session #1

• Qualitative Research training

o What is research?

o Difference between qualitative and quantitative

o Different interview methods including virtual interview methods

o Group exercise (simple interview exercise)

• Boundaries in interviewing

• Safety and support procedures for participants/Safety in the interview

**Training Session 3:** Interview questions Development

Date/Time: TBD

**Topics Covered:**

- Ice breaker
- Qualitative interview questions development (Stage 2)
- Practice interview questions in breakout rooms
- Debrief - Group discussion

**Training Session 4:** **Share the qualitative research findings**

Date/Time: TBD

**Topics Covered:**

- Ice breaker
- Share the qualitative research findings
- Open discussion: how to use these findings in the development of mobile app prototype
- Discuss the participants preference for a mobile app.
- Debrief

**Training Session 5 & 6: Share the mobile app prototype**

Date/Time: TBD

**Topics Covered:**

- Ice breaker
- Share the mobile app prototype with AAG participants
- Open discussion and feedback on the prototype
- Debrief - Group discussion

**Training Session 7: Share the refined mobile app prototype**

Date/Time: TBD

**Topics Covered:**

- Ice breaker
- Share the revised mobile app prototype with AAG participants
- Open discussion and feedback on the prototype
- Debrief - Group discussion

**Training Session 8: AAG Engagement Process Evaluation**

Date/Time: TBD
