# Supplementary material for: Co-designing a Sexual Health App With Immigrant Adolescents: Protocol for a Qualitative Community-Based Participatory Action Research Study
Source: JMIR Res Protoc. 2023 Mar 22;12:e45389. doi: 10.2196/45389 (PMC10131995; doi:10.2196/45389)
Supplement: Multimedia Appendix 2 [file resprot_v12i1e45389_app2.docx]

**Supplementary file 2**

**DEPICT steps, roles and guiding questions**

| **DEPICT step** | **Coordination functions** | **Team member roles** | **Questions to ask** |
| --- | --- | --- | --- |
| Dynamic reading | Collate, assign and distribute a subset of transcripts to each team member. Set deadlines and meeting times. | Review a subset of assigned transcripts. Record notes on important concepts. | What ideas seem to be important in these texts? (inductive) |
| Engaged codebook development | Assemble supplies (e.g. post-it notes, pens) and arrange for team meetings. Ensure skilled meeting facilitation. Ensure that a preliminary codebook is developed. Coordinate pilot testing and refining of codebook. | List important ideas for categorizing data. As a group, organize categories into clusters. Come to consensus around a preliminary codebook. Participate in pilot testing. | What is our agreed upon list of categories and sub-categories that we will use for our codebook? Do we have the right categories? Do we all understand what they mean and how to apply them? Do any require further refinement? |
| Participatory coding | Assign and distribute a subset of transcripts for coding to each team member. Set deadlines and meeting times. Provide training and support for novices. Coordinate a strategy for managing the data. | Review and code each assigned transcript. Return coding work to coordinator (in paper or electronic form). | Which sections of the transcript fit into which categories of our codebook? |
| Inclusive reviewing and summarizing of categories | Generate a list of quotes associated with each category. Assign team members a sub-set of categories to summarize. Distribute guiding worksheets for summarizing categories. | Work alone or in pairs to develop category summaries. Return work to the coordinator. | What are the main ideas? Where is there disagreement? What are some key quotes? Are there silences worth noting? What else is important to note that might help in the analysis of the larger project? |
| Collaborative analyzing | Arrange for one or more team meetings. Ensure skilled meeting facilitation. Select a note-taker in advance. Prior to meeting disseminate summaries for review. Ensure that consensus is reached and recorded on new understandings of the data. | Review summaries prior to meeting. Participate in a collaborative meeting to make sense of data. Graphically depict or create a figure that illustrates findings. Come to consensus on new understandings emerging from the data and what needs to be shared. | What does it all mean? What were our most important findings? What do we need to share and with whom? What questions do we still have? For critical analyses, what structural factors may help us understand why people chose to tell us the stories they shared (e.g. homophobia, neoliberalism). |
| Translating | Arrange for team meeting(s). Ensure skilled meeting facilitation Circulate meeting report with clear action items. | Develop a knowledge translation and exchange plan for sharing research results to all relevant stakeholders. Create a plan for equitably distributing this work. | Who needs to know what? How do they need to hear it? Who are the best messengers? How do we get the word out? Who on our team will be responsible for what and by when? |
