# Supplementary material for: Co-designing a Sexual Health App With Immigrant Adolescents: Protocol for a Qualitative Community-Based Participatory Action Research Study
Source: JMIR Res Protoc. 2023 Mar 22;12:e45389. doi: 10.2196/45389 (PMC10131995; doi:10.2196/45389)
Supplement: Multimedia Appendix 3 [file resprot_v12i1e45389_app3.pdf]

Review Type/Type d'évaluation: Committee Member 1/Membre de comité 1
Name of Applicant/Nom du chercheur: Meherali, Salima Moez
Application No./Numéro de demande: 459755
Agency/Agence: CIHR/IRSC
Competition/Concours: 2021-02-09 Operating Grant: Early Career Investigator Grants in Maternal, Reproductive, Child & Youth Health/Subv. de fonct.: Subventions de chercheurs en début de carrière en santé maternelle, en santé reproductive, et en santé des enfants/adolescents
Committee/Comité: Operating Grant: New Investigator Grants in Maternal, Reproductive, Child & Youth Health/Sub. de nouveaux chercheurs en santé maternelle, santé reproductive,et enfants/adolescents
Title/Titre: Co-designing a mobile application with immigrant adolescents for better sexual and reproductive health

---

Assessment/Évaluation:

The overarching research question is: What are the sexual and reproductive health (SRH) knowledge, information needs, and priorities of immigrant adolescents in Canada? The specific objectives are: 1. To better understand immigrant adolescents’ information, educational support and service access needs related to SRH. 2. To engage immigrant adolescents in the development of an interactive mobile sexual health promotion app. & 3. To evaluate the usability and effectiveness of mobile health or mHealth (the use of mobile phones to improve health) intervention. There is also a KT objective - To mobilize the research outcomes to a variety of stakeholders (such as immigrant adolescents, immigrant service agencies, etc.).

Despite the high level of SRH information consumption via mobile apps, evidence of the successful use of SRH
apps in Canada is limited, and apps that can increase uptake of SRH services and tools are nonexistent. This research
project will lead to the development of a demand-driven, culturally relevant, and easy-to-use mobile app to enhance
the uptake of evidence-based SRH information and services among immigrant adolescents in Canada. Targeting
adolescents and using engaging modalities has strong potential to effectively increase their involvement in SRH care
decision making, expand efficiencies in SRH care utilization, and ultimately improve adolescents’ SRH outcomes.

The background is well-justified, the theoretical framework generates excellent internal consistency, and the objectives and methods are appropriate and flow from each other. The user-centered design is well-articulated, and the methodological decisions, including recruitment sites, well-justified. I was curious about whether there would be opportunity for multi-lingual development within the app, as I did not see that planned or addressed. Potential challenges and mitigation strategies were also well-articulated. This project has substantial potential for impact.

Review Type/Type d'évaluation: Committee Member 2/Membre de comité 2
Name of Applicant/Nom du chercheur: Meherali, Salima Moez
Application No./Numéro de demande: 459755
Agency/Agence: CIHR/IRSC
Competition/Concours: 2021-02-09 Operating Grant: Early Career Investigator Grants in Maternal, Reproductive, Child & Youth Health/Subv. de fonct.: Subventions de chercheurs en début de carrière en santé maternelle, en santé reproductive, et en santé des enfants/adolescents
Committee/Comité: Operating Grant: New Investigator Grants in Maternal, Reproductive, Child & Youth Health/Sub. de nouveaux chercheurs en santé maternelle, santé reproductive,et enfants/adolescents
Title/Titre: Co-designing a mobile application with immigrant adolescents for better sexual and reproductive health

---

Assessment/Évaluation:

Summary:

This study uses an integrated knowledge translation and participatory action research approach to develop an interactive sexual health promotion mobile app that is specifically targeted with the needs of immigrant adolescents in mind. Four clear objectives are outlined: (1) To better understand immigrant adolescents' information, educational support and service access needs related to sexual and reproductive health (SRH); (2) To engage immigrant adolescents in the development of an interactive mobile sexual health promotion app; (3) To evaluate the usability and effectiveness of mobile health or mHealth (the use of mobile phones to improve health) intervention; (4) To mobilize the research outcomes to a variety of stakeholders (such as immigrant adolescents, immigrant service agencies, etc.). The applicants highlight how the needs for knowledge on SRH are not met, particularly for immigrant adolescents, and that knowledge gained can have long-term implications and importance for adolescents. The importance of the development of such a mobile app is also highlighted to be particularly relevant for adolescents, but also in the context of the pandemic where in-person care has been much limited. The nominated PA and team members have conducted a pilot study to better understand the integration challenges of South Asian immigrant adolescents in Alberta.

This is a resubmission of a previously submitted application. The applicants have addressed the majority of comments raised in the previous reviews including expanding the focus to include various immigrant backgrounds.

It is noted that the development of the mobile app will take place over a 9 month period (year 1-2) and then its usability will be tested over the period of 1 year from Year 2-3.

Strengths:
This is a really nicely written application that addresses a relevant health issue with implications for many Canadian youth and families. It is nicely structured and feasible. The team provides letters of support from different organizations, partner youth as well as matched funds from their main affiliated institutions. The team also outlines mitigation strategies to deal with certain limitations (eg. certain partners providing a voice for those who might not feel comfortable speaking, including the possibility of writing their responses in case participants might not feel comfortable given that potential presence of someone in the home when they are participating).

**Review Type/Type d'évaluation:**	Committee Member 2/Membre de comité 2
**Name of Applicant/Nom du chercheur:**	Meherali, Salima Moez
**Application No./Numéro de demande:**	459755
**Agency/Agence:**	CIHR/IRSC
**Competition/Concours:**	2021-02-09 Operating Grant: Early Career Investigator Grants in Maternal, Reproductive, Child & Youth Health/Subv. de fonct.: Subventions de chercheurs en début de carrière en santé maternelle, en santé reproductive, et en santé des enfants/adolescents
**Committee/Comité:**	Operating Grant: New Investigator Grants in Maternal, Reproductive, Child & Youth Health/Sub. de nouveaux chercheurs en santé maternelle, santé reproductive,et enfants/adolescents
**Title/Titre:**	Co-designing a mobile application with immigrant adolescents for better sexual and reproductive health

Assessment/Évaluation:

Applicants: The nominated principal applicant is Dr. Meherali (University of Alberta) and Dr. Munro (University of British Columbia) are strong applicants. They are also supported by an excellent multidisciplinary team with relevant experience in the field. The Nominated PI is experienced in the field (PhD in nursing from the University of Alberta and since 2018 holds an appointment as assistant professor in nursing at University of Alberta). and has a very strong track record including 31 publications and a number of grants as PI as well as co-applicant.

Weaknesses:

1. While the applicants write a nice paragraph in how sex and gender are addressed in the proposal, it is not clear how this will be taken into account in the actual study. Will focus groups also be run separately for different groups of adolescents based on their sex/gender? Perhaps they might feel less comfortable speaking about these issues with someone of another sex. While the applications mention that separate groups will be run for adolescents in two larger age ranges (eg. 12-16 years vs. 16-19 years), it is not explicitly addressed whether or how groups will deal with different sexes/genders.

2. Further, while the team mention that immigrant adolescents in 3 large Canadian cities (Toronto, Edmonton and Vancouver) will participate, it is not clear exactly how these adolescents will be recruited (they indicate through some different channels) and whether information on their immigration status will be taken into account. For instance, whether these are first generation immigrants vs. second generation, and whether they will study the contribution of this or other factors such as the age at which they arrived in Canada (eg. very recent immigrant versus arriving as a young child, or second generation).

Budget -total cost $69,414 -overall, budget seems reasonable with some costs pertaining to mobile app development (20k), personnel costs (including student RAs).

Review Type/Type d'évaluation: Committee Member 3/Membre de comité 3
Name of Applicant/Nom du chercheur: Meherali, Salima Moez
Application No./Numéro de demande: 459755
Agency/Agence: CIHR/IRSC
Competition/Concours: 2021-02-09 Operating Grant: Early Career Investigator Grants in Maternal, Reproductive, Child & Youth Health/Subv. de fonct.: Subventions de chercheurs en début de carrière en santé maternelle, en santé reproductive, et en santé des enfants/adolescents
Committee/Comité: Operating Grant: New Investigator Grants in Maternal, Reproductive, Child & Youth Health/Sub. de nouveaux chercheurs en santé maternelle, santé reproductive,et enfants/adolescents
Title/Titre: Co-designing a mobile application with immigrant adolescents for better sexual and reproductive health

Assessment/Évaluation:

Globally, Canada has the second-highest percentage of immigrants (21.9%). Of all these newcomers to Canada, approximately 34% are under the age of 25 years with over 60 % of immigrant youth (15–24 years) coming from Asia, the Middle East, and Africa. Understanding healthcare service information needs and utilization by immigrant adolescents is vital for thorough health planning, policy, and healthcare delivery. Neglect of specific adolescents sexual and reproductive health (SRH) needs can pose serious challenges and affect physical and mental health, future employment, economic wellbeing, and adolescents' ability to reach their full potential. A growing body of literature suggests that immigrant adolescents lack SRH knowledge, and use fewer sexual health services and sex education resources than non-immigrant adolescents. Innovative ways to deliver sexual health information are more important than ever before due to the impact of the COVID-19 pandemic on access to SRH services globally. Interruptions in SRH information and service delivery have a serious impact on vulnerable adolescent's health and well-being. Amidst the pandemic, technology has largely provided the means to support a recalibration of health systems, service provision, and information delivery. Today it is easier than ever before to access SRH information from digital media. Smartphone applications have been shown to be highly effective in providing health information to adolescents. The overarching purpose of this community based participatory action research is to actively engage with immigrant adolescents to develop, implement, and evaluate mobile health or mhealth intervention (i.e., mobile app) to improve SRH information of immigrant adolescents in Canada. This research project will be conducted in four stages structured based on user-centered design principles. Stage 1 Empathize: Recruit, convene, and train three adolescents' advisory groups (in Edmonton, Toronto, and Vancouver) (June 2021- Dec 2021). Stage 2 Define and Ideate: Explore SRH information and service needs through focus groups (FGs) with immigrant adolescents (3–4 FGs in each city, total n=60–80) (Jan 2022–June 2022). Stage 3 Prototype: Collaborate with mobile developers to build and iteratively design the app with support from adolescent advisors (July 2022–Dec 2022). Stage 4 Test: Return to focus group settings to share the app prototype, gather feedback on usability, refine, and release the app (March 2023–March 2024).

This project builds on well-established relationships between researchers, clinicians, immigrant service agencies and communities of immigrant adolescents. The interdisciplinary team includes experts in SRH, nursing, gender, and environment studies, computing science, population and public health, community-based research as well as knowledge end-users in SRH information dissemination and health care policy. This will have the potential to advance the limited knowledge base on SRH knowledge and information needs of immigrant adolescents in Canada. This study will address the gaps by exploring the SRH priorities, health information needs, and developing innovative strategies to improve the SRH of immigrant adolescents. Engaging adolescents throughout the study will increase their involvement in SRH care decision-making, expand efficiencies in SRH care utilization, and ultimately improve adolescents' SRH outcomes. The model/app we develop will be transferable to other adolescent groups, is scalable in international contexts, and simultaneously leverages significant economies-of-scale.

Review Type/Type d'évaluation: Committee Member 3/Membre de comité 3
Name of Applicant/Nom du chercheur: Meherali, Salima Moez
Application No./Numéro de demande: 459755
Agency/Agence: CIHR/IRSC
Competition/Concours: 2021-02-09 Operating Grant: Early Career Investigator Grants in Maternal, Reproductive, Child & Youth Health/Subv. de fonct.: Subventions de chercheurs en début de carrière en santé maternelle, en santé reproductive, et en santé des enfants/adolescents
Committee/Comité: Operating Grant: New Investigator Grants in Maternal, Reproductive, Child & Youth Health/Sub. de nouveaux chercheurs en santé maternelle, santé reproductive,et enfants/adolescents
Title/Titre: Co-designing a mobile application with immigrant adolescents for better sexual and reproductive health

---

Assessment/Évaluation:

The candidate is an Assistant Professor at University of Alberta. The candidate is the PI on developing adolescent advisory groups to inform sexual and reproductive health research for immigrant adolescents in Canada funded by the Women and Child Health Care Institute, has established an adolescent advisory group in Edmonton that inventively informs research, and has developed a Community-Based Girls' Empowerment Intervention to empower young immigrant girls (age 12-19 years) in Canada which included a 10-week's Girls' Voice curriculum. A peer mentorship program was created at the end of this curriculum implementation. The candidate also developed and augmented an innovative knowledge translation tool for parents, in which a whiteboard video and infographic on acute otitis media for Canadian parents and then augmented the tools in a different cultural context to evaluate the scalability of the tools. The Whiteboard video title "Mom! My ear hurts: What to do when your child has ear pain" received a special commendation in the 2018 IHDCYH talk video competition. The tool was revised and translated into Urdu and now available on the YouTube platform.

In the proposal, for Stage 3 (Prototype), collaboration with mobile developers to build and iteratively design the app with support from adolescent advisors (July 2022–Dec 2022) was mentioned. However, in the partner section (or elsewhere of the package), no support letter from mobile developers was provided. For the successful development and maintenance of a smartphone app, academic alone may not be enough, and active participation by private sector (e.g., IT company or carrier) may be crucial. For apps developed specifically for immigrants, language and cultural differences need to be taken into consideration at each stage of development, and inputs from immigration groups in the early stage of development may be needed, not just after the English version has been created and released.
